# Predictors of recovering ambulation after hip fracture inpatient rehabilitation

**DOI:** 10.1186/s12877-018-0884-2

**Published:** 2018-08-31

**Authors:** Francesca Cecchi, Silvia Pancani, Desiderio Antonioli, Lucia Avila, Manuele Barilli, Massimo Gambini, Lucilla Landucci Pellegrini, Emanuela Romano, Chiara Sarti, Margherita Zingoni, Maria Assunta Gabrielli, Federica Vannetti, Guido Pasquini, Claudio Macchi

**Affiliations:** 1 Don Carlo Gnocchi Foundation, IRCSS, Via di Scandicci, 269, 50143 Florence, Italy; 2 Department of Experimental and Clinical Medicine, University of Florence, Florence, Italy

**Keywords:** Hip fracture, Rehabilitation outcome, Predictors of ambulation

## Abstract

**Background:**

Despite progress in surgery and care, hip fracture (HF) remains a catastrophic event, burdened with high risk of mortality and disability. This study aims at identifying predictors of recovering ambulation after intensive inpatient rehabilitation within the Tuscany Region HF rehabilitation pathway.

**Methods:**

All HF patients referred from acute care to the two Massa-Carrara Rehabilitation facilities January 2015–June 2017 were enrolled. Comorbidity Total Score (CIRS) defined high- or low-care setting referral. Recovery of ambulation, with or without aid, (assessed by SAHFE) was the primary outcome. Personal data, comorbidity, cognitive (MMSe) and pre-fracture function (mRANKIN) were recorded on admission. Outcomes included hospital readmission, length of stay (LOS) and home discharge. Urinary catheter, bedsores, disability (modified Barthel Index-mBI), communication disability (CDS), trunk control (TCT), pain (NRS), and ambulation were recorded (admission-discharge).

**Results:**

Of 352 patients enrolled (age 83.9 ± 7.1; 80% women), 1 died and 6 were readmitted to acute-care hospital; 97% patients referred to high-care, and 64% referred to low-care, presented moderate-high comorbidity on admission. Median LOS was 22 days; 95% patients were discharged back home; daily functional gain (mBIscore/LOS) was 1.3 ± 0.7. Patients who recovered ambulation on discharge were 84%. Older age, higher comorbidity, bladder catheter, impaired trunk control, worse cognitive and functional status on admission, and pre-fracture disability were associated to poor outcome, but only higher comorbidity and impaired communication on admission predicted failure to recover ambulation on discharge.

**Conclusion:**

In HF patients entitled to intensive inpatient rehabilitation, moderate-high comorbidity and impaired communication are frequent findings and predict rehabilitation failure.

## Background

The event of hip fracture (HF) in elderly persons, a continuously growing segment of the general population [1, 2], increases exponentially with age, with a worldwide annual incidence expected to reach 4.5 million by 2050 [3, 4]. HF often selects patients defined as frail [5], characterized by high complexity and high risk of incident disability or worsening of existing disability. Indeed, despite progress in surgery and clinical care, HF is still a catastrophic event, burdened with a high risk of mortality and residual disability: approximately one-third of HF patients are institutionalized, and half experience a permanent post-fracture disability [6]. Identifying therapeutic pathways that ensure maximum functional recovery, while containing direct and indirect HF costs, is therefore of vital importance.

Rehabilitation is crucial for the functional recovery of elderly patients with HF [7, 8]. The optimal setting, intensity, and duration of rehabilitation are current issues of debate, but the evaluation of rehabilitation performance is often confined to the variation of a single functional scale, undervaluing the contribution of care processes and of clinical complexity and nursing needs to a final functional outcome [9]. Furthermore, practice is still inconsistent across different facilities [10].

In the Italian Tuscany region, in 2008, 7027, persons aged 65 and over were hospitalized for hip fracture, 1551 (22.1%) men and 5476 (77.9%) women, with 7.8 admissions per 1000 Tuscan residents over 64 (IC95% 7.7–8.0), 9.7 ‰ IC95% 9.4–10.0 women and 4.8 ‰, IC95% 4.6–5.1 men [11]. Based on these alarming data, the Regional Council appointed a panel of experts to develop an evidence-based HF clinical–rehabilitation pathway [7, 8], published in Tuscan regional Council resolution N 677 of 30-07-2012 and implemented thereafter. Recent data from 2017, confirm the impressive number of hip fractures affecting persons aged 65 and over in Tuscany: 7665 persons, 1992 man (26%), 5673 women (74%). The resolution stated appropriateness criteria for different rehabilitation settings and average rehabilitation intervention duration foreseen for each setting [12], thus ensuring homogenous standards for HF rehabilitation in Tuscany.

The purpose of this study is to prospectively identify predictors of failure to recover ambulation in HF patients from the Massa-Carrara area, who received inpatient intensive rehabilitation, according to the Tuscany Region pathway.

## Methods

### Regional resolution

The pathway defines appropriate timing of surgery (48 h from fracture) and to hospital discharge (4 days from surgery if the patient is clinically stable). Patients aged 65 and over who are unable to walk with aid, though allowed weight bearing, are referred to intensive inpatient rehabilitation; Length of Stay (LOS) is considered appropriate within 21 days [12]. Comorbidity is scored by the Cumulative Illness Rating Scale, Total Score (CIRS-TS), scoring both number of diseases (14 items) and severity of chronic comorbidity and of complications, range 13–69 [13]. Patients with moderate-high comorbidity (CIRS ≥ 19) are referred high-care intensive rehabilitation, while those with CIRS< 19 to low-care. In both cases, patients should be fit to at least 3 h of rehabilitation per day. Non-weight bearing patients are discharged home or to intermediate care until weight bearing is allowed. Cognitive impairment is screened by the Mini Mental State examination, range 30 best- 0 worst (MMSe, [14]); severely demented patients, scoring MMSe≤ 10, and previously non-ambulating patients (mRANKIN pre-fracture disability) are entitled to home counseling, and patients able to use a walker on discharge, with adequate home caregiving, to outpatient rehabilitation [7, 8].

### Setting

The Rehabilitation facilities of Marina di Massa and Fivizzano, Fondazione Don Gnocchi, provide rehabilitation for all the Massa-Carrara area: Massa has 19 high-care and 5 low-care beds, Fivizzano 13 high-care and 19 low-care beds. High clinical care setting requires daily vital parameter and medical assessment, and 24 h MD availability.

### Inpatient rehabilitation

Inpatient intensive rehabilitation, coordinated by a specialist in Physical and Rehabilitation Medicine, implies individualized interdisciplinary care [15], delivered by a team of physiotherapists, occupational therapists, nurses, social health professionals, psychotherapists, always including the patient and his/her caregiver. Physiotherapy, tailored to individual needs, aims at balance recovery, muscle strengthening, hip range of motion, pain control, and ambulation (from walker to cane, to no aid, when possible); occupational therapy is focused on activities of daily living training. Pain is routinely monitored, and treated, either pharmacologically or by physical/instrumental therapy. Psychological support is available for patients and caregivers. Technical advice on aids for autonomy and eventual home adjustments is provided. Neuropsychological/logopedics assessment and treatment is available if undiagnosed cognitive decline/dysphagia/aphasia are suspected. The program engages each patient for an average of 3 h per day 6 days a week and is periodically redefined throughout the stay.

### Patients

From January 1st, 2015 to June 30th, 2017, all HF patients referred to the Don Gnocchi Rehabilitation facility of Massa and Fivizzano by the local public health specialists were consecutively enrolled. All patients provided written consent to anonymous treatment of their personal and clinical data. As this was notified by Don Gnocchi Foundation to the Ministry of Health as a prospective observational study, in agreement with national guidelines, no formal ethics approval was required (MD 20 March G.U. n. 76 del 31-3-2008, GU n76, 31/03/2008).

### Measures

Outcomes included hospital readmission, death, length of stay (LOS) and a series of functional and clinical parameters. Personal data, comorbidity (CIRS), cognitive level (MMSe), and pre-fracture functional level, modified Rankin scale (mRankin, [16]), ranging 0-no symptom - 5-severe disability, were collected on admission. According to a comprehensive approach to rehabilitation quality assessment [17], rehabilitation outcomes included both functional and clinical parameters, recorded on admission and on discharge: urinary catheter, bedsores, ADL disability by the modified Barthel Index (mBI, [18]) ranging 100-no to 0-complete disability, disability in communication, by the Communicative Disability Scale (CDS, [19]), ranging 0-complete to 4-no disability in communication [19], trunk control, by the Trunk Control Test (TCT, [20]), ranging 0-no to 100-complete control, Pain, by the Numeric Rating Scale (NRS)-PAIN, ranging 0-no to 10-worst possible pain [20], and ambulation, by the Standardized Audit of Hip Fracture In Europe (SAHFE) [21]. The primary rehabilitation outcome was SAHFE, scoring 1-Independent outdoor ambulation, without help, 2-Outdoor ambulation with aid, 3-Indoor ambulation with aid (except walker), 4-Indoor ambulation with a walker, 5-Unable to walk/ using a wheelchair.

### Statistical analysis

All statistical analyses were performed using IBM SPSS Statistics 23.0 for Windows [22]. Clinical and functional scores on admission and on discharge were compared. CIRS, TCT and mBI scores were compared through a paired t-test. A Wilcoxon signed rank test was used to compare NRS, CDS and SAHFE scores, while dichotomous variables were analyzed using a McNemar test.

Patients that recovered ambulation on discharge (“able to walk”, SHAFE< 5) were compared to those who did not (“unable to walk”, SAHFE = 5. Patients with missing data on discharge (*n* = 28) were excluded from further analysis. Of the remaining 324 patients, age and CIRS, Barthel, TCT and MMSe scores were compared by an independent t-test, while a Mann Whitney U test was used to analyze mRankin, CDS, and NRS scores. Gender, use of bladder catheter and presence of bedsores were compared using a Chi-Square test.

In a logistic regression analysis, parameters shown to be significantly different between the two groups (“able to walk” and “unable to walk”) were considered independent variables. The recovery of walking function was used as a dependent variable while age and gender were assumed as confounding factors.

In all the analyses, level of significance was set at a *p*-value< 0.05.

## Results

All 352 HF patients (80% women, 20% men; 318 low-, 34 high-care) accessing the two rehabilitation facilities were enrolled in the study. Characteristics of the studied sample are resumed in Table 1. Among them, one patient (0.003%) died, 4 (0.01%) were discharged to resident care, 6 (0.02%) were readmitted to an acute care Hospital, 6 (0.02%) were referred to intermediate care and 1 (0.003%) was referred to a different rehabilitation facility; the remaining 334 patients (95%) were discharged back home. Patients’ mean age was 83.9 ± 7.1 years (range: 65–99 years) while LOS showed a median value of 22 days (IQR: 7 days).

**Table 1:** Patients’ characteristics, clinical and functional scores measured at admission and discharge

Parameters	N	Admission		Discharge		*p*-value
		Mean Median	Std. Dev. (Interquartile Range)	Mean Median	Std. Dev. (Interquartile Range)	
		Percentage		Percentage		
Age (years)	352	83.9	7.1			
Gender (F %)	352	80%				
Hospitalization period (days)	352	22	(7)			
MMSe at admission (score)	266	19.6	7.1			
RANKIN	352	2.0	1.3			
CIRS	352	21.0	3.8	20.9	3.7	0.255
CDS	349	4	(0)	4	(0)	0.239
SAHFE	324	=5.0		3.9	0.7	< 0.001
Barthel index	352	29.5	17.8	58.7	22.9	< 0.001
TCT	352	41.2	24.8	68.2	23.1	< 0.001
NRS	345	3	(6)	0	(2)	< 0.001
Urinary catheter (%Y)	349	23%		10%		< 0.001
Bedsore (%Y)	352	33%		19%		< 0.001

Parameters characterized by a normal distribution are expressed as mean and standard deviation, parameters with a non-normal distribution as median and interquartile range, dichotomous parameters as a percentage

*MMSe* mini-mental state examination, *CIRS* cumulative illness rating scale, *CDS* communication disability scale, *SAHFE* Standardized Audit of Hip Fracture in Europe, *TCT* trunk control test, *NRS* numeric rating scale

Only 1 of 34 patients was referred to high care with CIRS< 19, while 226/318 (71%) patients referred to low care presented CIRS≥ 9. For the latter, clinical and nursing care were delivered according to high clinical care requirements. Patients with MMSe< 10 were 28 (8%); 50% of patients presented some degree of cognitive impairment (MMSe score < 21). As to pre-fracture disability, 74 (21%) reported moderate, 51 (14.5%) moderately severe, and 10 (2.8%) severe disability (bedridden before fracture); 96 patients presented some degree of impairment in communication on admission (27.5%).

Table 1 shows also the comparison between patients’ characteristics and clinical and functional scores measured on admission and on discharge. SAHFE, mBI, TCT and NRS scores were significantly improved. Significantly fewer patients had urinary catheter or bedsores on discharge: Average mBI delta score was 29.0 ± 14.7, that, divided by LOS provided a rehabilitation efficiency rate of 1.3 ± 0.7.

Characteristics of the patients excluded from the second analysis are reported in Table 2. Among the excluded patients 1 died, 6 were referred to an ER and the remaining had missing data on discharge in one or more scales. Excluded subjects were significantly different from those included in the further analysis in terms of older age (*p* = 0.001), lower MMSe (*p* = 0.048), mRankin (*p* = 0.035), CIRS and SDC scores (both at admission and discharge, *p* < 0.001) and SAHFE score at discharge (*p* < 0.001). They presented also a significantly higher percentage of bladder catheters both at admission and discharge (*p* = 0.002 and *p* < 0.001, respectively) and a higher number of bedsores at discharge (*p* = 0.001).

**Table 2:** Characteristics of patients (*N* = 28) excluded from second analysis. Clinical and functional scores measured at admission and discharge

Parameters	N	Admission		N	Discharge	
		Mean Median	Std. Dev. (Interquartile Range)		Mean Median	Std. Dev. (Interquartile Range)
		Percentage			Percentage	
Age (years)	28	88.1^*^	6.9			
Gender (F %)	28	75%				
Hospitalization period (days)	28	21.5	(13)			
MMSe (admission score)	14	16.0^*^	5.3			
RANKIN	28	2.6^*^	1.3			
CIRS	28	23.4^*^	3.3	28	23.6^*^	3.6
CDS	28	3^*^	(1)	25	3^*^	(1)
SAHFE	5	=5.0		15	4.9^*^	0.3
Barthel index	28	23.0	14.3	21	47.2	22.9
TCT	28	32.6	22.0	21	61.5	22.7
NRS	28	3	(4)	22	0	(1)
Urinary catheter (%Y)	28	46%^*^		28	39%^*^	
Bedsore (%Y)	28	43%		28	43%^*^	

Parameters characterized by a normal distribution are expressed as mean and standard deviation, parameters with a non-normal distribution as median and interquartile range, dichotomous parameters as a percentage

*MMSe* mini-mental state examination, *CIRS* cumulative illness rating scale, *CDS* communication disability scale, *SAHFE* Standardized Audit of Hip Fracture in Europe, *TCT* trunk control test, *NRS* numeric rating scale

^*^significantly different (*p* < 0.005) compared to patients included in the second analysis

Table 3 shows ambulation according to SAHFE achieved upon discharge: 84% patients recovered ambulation, and about 7% were able to walk outdoors on discharge.

**Table 3:** number [percentage] of patients presenting with SAHFE score = 5 at admission and with different SAHFE score at discharge

SAHFE score at discharge	Nr of patients [%]
1 Able to walk autonomously outdoor without aid	1 [0.3%]
2 Able to walk outdoor with aid	21 [6.5%]
3 Able to walk indoor with aid (except walker)	33 [10.2%]
4 Able to walk indoor with a walker	218 [67.3%]
5 Unable to walk, use of wheelchair	51 [15.7%]

As can be seen from Table 4, patients “unable to walk” (SAHFE = 5 on discharge, *n* = 51, 16%) were significantly older and showed significantly worse CIRS, RANKIN, CDS, mBI, TCT and MMSe scores and more of them needed a urinary catheter, while no significant differences were found in gender, NRS-pain score, presence of bedsores and LOS compared to those “able to walk” on discharge (SAHFE< 5, *n* = 273, 84%).

**Table 4:** Parameters assessed at admission for those patients that recovered walking independence (able to walk) and those that did not (unable to walk)

Parameters assessed on admission	Able to walk (*n* = 273)		Unable to walk (*n* = 51)		*p*-value
	Mean Median	Std. Dev. (IQR)	Mean Median	Std. Dev. (IQR)	
	Percentage		Percentage		
Age (years)	83.2	7.1	85.3	6.6	0.055
Gender (%F)	80%		81%		0.974
CIRS (score)	20.6	3.6	23.2	4.1	< 0.001
RANKIN (score)	1.9	1.3	2.5	1.4	0.006
CDS (score)	3.7	0.7	3.3	0.8	< 0.001
mBI (score)	31.2	17.5	20.8	16.9	< 0.001
TCT (score)	43.8	23.8	29.8	27.1	< 0.001
NRS (score)	3	(6)	2	(4)	0.248
Bladder catheter (%Y)	17%		40%		< 0.001
Bedsore (%Y)	32%		36%		0.542
Hospitalization period (days)	22	(7)	22	(10)	0.854
MMSe (score)	20.3	7.0	17.2	7.1	0.015

Parameters characterized by a normal distribution are expressed as mean and standard deviation, parameters with a non-normal distribution as median and interquartile range (IQR), dichotomous parameters as a percentage

*CIRS* cumulative illness rating scale, *CDS* communication disability scale, *mBI* modified Barthel index, *TCT* trunk con-trol test, *NRS* numeric rating scale, *MMSe* mini-mental state examination

Table 5 shows the results for the logistic regression: comorbidity (CIRS, *p* = 0.001) and disability in communication (CDS, *p* = 0.013) were independently associated with failure to recover ambulation. The model was statistically significant (*p* < 0.001) and explained the 24% of the variance in the dependent variable.

**Table 5:** Association between parameters measured at admission and ability to recover ambulation according to a Logistic Regression model

	B	S.E.	Sig.	Exp(B)	Nagelkerke R Square
Model			< 0.001		0.241
Constant	3.297	3.459	.341	27.023	
CIRS	−.175	.053	.001	.839	
RANKIN	−.035	.174	.839	.965	
CDS	.755	.303	.013	2.129	
Mbi	−.002	.018	.893	.998	
TCT	.018	.010	.088	1.018	
Urinary catheter	.778	.450	.084	2.177	
Gender	−.183	.536	.733	.833	
Age	−.029	.033	.382	.972	
MMSe	.045	.035	.197	1.047	

## Discussion

Traditional methods of evaluation of rehabilitation performance are mainly focused on the admission-discharge variation of a disability scale score. In evaluating predictors of HF rehabilitation failure, we chose to consider both clinical and functional parameters, in the attempt to investigate the complex relationship that binds care needs and patient characteristics to final functional gain.

We verified that cognitive and pre-function referral criteria were aligned to pathway requirements: only 28 patients presented an MMSe score ≤ 10 on admission and only 10 (2.8%) were already unable to walk before fracture. On the contrary, we found a substantial discrepancy in the comorbidity index: while only 1 patient with low comorbidity entered high-care rehabilitation, up to 64% of “low-care” patients presented a moderate-high comorbidity score. In the specific cases, the clinical decision was to deliver high care, regardless of referral, but this is not always possible in clinical practice. From a broader perspective, we hypothesize that if an older, previously ambulating HF patient, does not recover walking with aid on the 4th–5th day after surgery, this is very likely to be accounted by either diagnosed or undiagnosed comorbidity, that must be addressed in the rehabilitation process. So, as our results suggest, we argue that all these geriatric patients should be regarded as possibly complex and referred to high-care rehabilitation settings [23].

The rehabilitation program was overall highly successful: we reported 1 death and 6 hospital readmissions, and only 6 patients were institutionalized, while 95% were discharged back home. Although median LOS was 1 day longer than that the pathway required, it was shorter than what reported in most similar studies (22 days vs 28–30) [6, 24–27]. We also found a significant reduction of pain, bedsores, and number of patients with urinary catheter on discharge: these conditions are rarely reported as HF rehabilitation outcomes, although they may impact on clinical complications as well as on functional gain [9, 28]. Improvement of mobility was high (29% mBI increase), and the daily functional gain (improve in functional score divided by LOS) was highly efficient [6, 25–27, 29]. Recovery of ambulation was also achieved by a very high percentage of patients (84%); a similar Korean study conducted on younger patients receiving on average 30 days of inpatient intensive rehabilitation, reported a 70% recovery [27].

We assessed rehabilitation outcome at the time of discharge from inpatient rehabilitation, although patients may have continued rehabilitation further. We chose this time as, on discharge, the patient returns to his/her natural environment and because inpatient rehabilitation accounts for the most relevant share of HF rehabilitation [25]. We could only collect data from patients referred to inpatient rehabilitation, and not from all HF patients in the Massa-Carrara area. This is the main limitation of our study, as we could neither evaluate the first part of the pathway (acute care and surgery) nor elaborate on what happened to those who were not referred to inpatient rehabilitation. Theoretically, this also led to a selection bias in the study population, but, actually, the characteristics of our sample were similar to Canadian, Israelian, Asian, and US studies [6, 25–27] as the pathway appropriateness criteria for referral were defined on evidence-based guidelines [7, 8].

Analysis of predictors of response to treatment may provide relevant insight into rehabilitation practice [30, 31]. In our analysis, patients who did not recover ambulation, with or without aid (16%), were significantly older and showed a significantly higher comorbidity, pre-fracture disability, disability in mobility and communication, worse trunk control, and reduced cognitive ability, compared to those that recovered ambulation; further, more of them needed a urinary catheter on admission, while LOS did not differ between the two groups. In the regression analysis, only comorbidity (CIRS) and disability in communication (CDS) remained independent predictors of failure to recover ambulation. As to this secondary analysis, we must mention the exclusion of 28 patients, either due to death/acute care readmission or missing data. However, these patients presented lower SDC and higher comorbidity on admission and overall worse outcome, thus suggesting that their exclusion was not a substantial bias to our final results, that are in line with some [9, 27], but not all studies focusing on HF recovery [25, 26, 32, 33]. Indeed, we must also point out that our model, although significant, explained only 24% of the variance of the final outcome; as we already acknowledged, the lack of information on the first stages of care and on surgery (timing, type) is the main limitation of our study, and, probably, our model would have been empowered by including this information [24, 27]. On the other hand, as generally happens in clinical practice, we could focus on what can be detected and addressed in the rehabilitation setting, regardless of what happened before admission. A possible reason for our finding that pre-fracture functional level and cognition did not predict ambulation on discharge, might be the exclusion, by pathway criteria, of the extremes of the spectrum (either of those already able to walk on hospital discharge and of those who were already severely disabled in mobility and/or cognition before the fracture), but, again, this cannot be verified without the data from patients excluded from inpatient rehabilitation after surgery.

Comorbidity on admission, including both chronic and acute complications, was a powerful predictor of HF rehabilitation outcome, confirming that, in our sample, the functional gain was strictly related to individual medical-nursing complexity [34]. This result indirectly supports our previously expressed view that HF patients require high standard clinical and nursing care to obtain the highest possible functional gain [35]. Bernardini et al. already showed in a large sample of inpatient rehabilitation patients, including HF, that those with clinical complexity on admission presented a lower functional recovery than those without clinical complexity [9]; Kim et al. [27] showed that pre-fracture ambulatory capacity and combined medical disease predicted ambulatory capacity with walking aid, whereas MMSe and functional status on admission did not.

While the predictive value of comorbidity to HF outcome is in line with most of the literature, the predictive value of communication skill is a rather novel, if not unexpected, finding. The main reason for this is probably the fact that communication is not routinely screened or considered in HF patients. In our sample, 30% patients presented an impairment in communication on admission; CDS score was significantly improved from admission to discharge. The CDS was designed for rapid screening of communication skills. Evaluation is carried out in an informal, but minimally structured communication setting (for example, during the collection of the anamnesis and the execution of the objective examination, during the assistance maneuvers made by the nursing staff), thus it is can be very rapidly and easily administered also to severely compromised patients, which cannot be evaluated with formal neuropsychological tests [19]. The CDS depends on verbal skills, sensory abilities, relationship, and collaboration. It scores disability, independent of its cause/causes (neuropsychological, sensory or psychomotor disorder), that must be addressed by specific diagnosis and treatment. We found that both MMSe and CDS were significantly associated to a worse outcome, but, in the regression analysis, only CDS predicted failure to recover walking ability. At least in this sample, excluding severely demented patients, cognitive impairment seemed not to impact rehabilitation outcome directly, but rather, if so, through reduced communication abilities. These findings confirm those reported by Mc Gilton and by Resnick [6, 36], supporting the appropriateness of referring cognitively impaired persons to intensive inpatient rehabilitation, while suggesting dedicating specific attention to communication strategies. Communication impairment by any cause (be it aphasia, depression, deafness or any other possible cause) may indeed compromise therapeutic alliance and learning abilities which deeply affect rehabilitation [19]; on the other hand, impaired communication may also indirectly affect rehabilitation outcome, as it may be a symptom of additional comorbidity. The good news is that many communication problems, stemming either from neurological, clinical, sensorial, cultural or psychological problems, may often be successfully addressed by either modifying patient factors, environmental factors or style of communication. Although we cannot make inference on how optimal management of comorbidity and impairment of communication may modify the final outcome of HF rehabilitation, the results of our study suggest that high care of clinical-nursing complexity and screening and address of any communication problems are recommended for all HF patients referred to intensive rehabilitation, to improve care provision and, possibly, final functional outcome.

## Conclusions

The outcome of HF patients referred to intensive inpatient rehabilitation was very successful, and the interdisciplinary rehabilitation provided proved to be highly efficient, with a 1.3 mBI score daily functional gain and 84% patients recovering ambulation on discharge. Moderate-high comorbidity was a frequent finding, also in patients referred to a low-care setting. Higher comorbidity and impairment in communication ability on admission predicted failure to recover ambulation, independent of pre-fracture disability, compromised functional status on admission, age and MMSe. These results support the importance of addressing nursing and clinical complexity and of screening, and eventually addressing, any-cause communication disability in HF rehabilitation.

## Abbreviations

ADL: Activities of daily living
CDS: Communicative disability scale
CIRS: Cumulative illness rating scale, total score
HF: Hip fracture
IQR: Interquartile range
LOS: Length of stay
mBI: modified Barthel Index
MMSe: Mini Mental State examination
NRS: Numeric rating scale
SAHFE: Standardized Audit of Hip Fracture In Europe
TCT: Trunk control test
TS: Cumulative final score
